# (Re)Configuring Hybrid Meetings: Moving from User-Centered Design to Meeting-Centered Design

**DOI:** 10.1007/s10606-020-09385-x

**Published:** 2020-11-19

**Authors:** Banu Saatçi, Kaya Akyüz, Sean Rintel, Clemens Nylandsted Klokmose

**Affiliations:** 1 Department of Digital Design and Information Studies, Aarhus University, Helsingforsgade 14, 8200 Aarhus N, Denmark; 2 Department of Science and Technology Studies, University of Vienna, Universitätsstraße 7/Stiege II/6. Stock (NIG), 1010 Vienna, Austria; 3 Microsoft Research, 21 Station Road, CB1 2FB Cambridge, UK

**Keywords:** Configuration, Conversation analysis, Hybrid meetings, Micro-analysis, User-centered design

## Abstract

Despite sophisticated technologies for representational fidelity in hybrid meetings, in which co-located and remote participants collaborate via video or audio, meetings are still often disrupted by practical problems with trying to include remote participants. In this paper, we use micro-analysis of three disruptive moments in a hybrid meeting from a global software company to unpack blended technological and conversational practices of inclusion and exclusion. We argue that designing truly valuable experiences for hybrid meetings requires moving from the traditional, essentialist, and perception-obsessed user-centered design approach to a phenomenological approach to the needs of meetings themselves. We employ the metaphor of ‘configuring the meeting’ to propose that complex ecologies of people, technology, spatial, and institutional organization must be made relevant in the process of design.

## Introduction

The ‘meetingisation’ of work stems from the increasing need for collective means of social orientation and coordination, especially in post-industrial economies (van Vree 2019). Work has also become more distributed, increasing the importance and variety of hybrid meetings, in which co-located and remote participants engage via audio and video technologies. While representational fidelity for remote participants has grown ever-more sophisticated, hybrid meetings often become stalled at the user-practice level due to combinations of technological problems and failures of design to support hybrid meeting dynamics.

To improve hybrid meeting technologies, we must better understand the special problems of meeting continuity that arise from hybridity itself. These problems are often revealed in practices of inclusion and exclusion of remote participants in hybrid meetings. By inclusion, we mean episodes in which the meeting continuity is disrupted either to organize the participation of a remote user, or to organize meeting tasks around a spatial orientation that accounts for both remote and local participants. By exclusion, we refer to episodes in which meeting continuity is itself organized to accountably exclude remote participants during or following a technological breakdown.

Our research question is: How do practices of inclusion and exclusion of remote participants arise in hybrid meetings, especially in the context of technology troubles? We know that when technology troubles arise in the video calls of long-distance relationships, couples aim to maintain conversational continuity rather than fixing technological problems (Rintel 2013). Couples treat technology trouble as an accountable part of continuity by explicitly blaming it, treating it as ignorable, or incorporating the trouble into their relational talk. They do so because it is the relationship, not the call, that is primarily at stake. In this study, we expected and found similar practices being deployed in the business context, with the addition of group, spatial, institutional, and technological dynamics.

Bødker (2015) argues that third-wave HCI research focused on the use of new private consumer technologies over the prior second-wave’s focus on how cooperation, learning and participation among multiple users is enacted through technological artifacts in the workplace. While the emphasis on ‘experience-based use situations’ has been positive, Bødker (2015, p. 26) argues that there has been some loss of a holistic understanding of design for adoption of technologies in their ecological context. Analogously, we propose that understanding how practices evolve in hybrid meetings requires moving from the individualistic actor-activity based approach to *communication in the meeting* to a phenomenon-centered approach to the *meeting itself*. In so doing, we blend two prior conceptual frameworks (Akrich 1992; Woolgar 1990) to suggest employing the metaphor of ‘configuring the meeting’, re-thinking how the complex ecologies of human and non-human actors, in multiplicities of meetings and their socio-material and socio-technical dynamics, can be made relevant in the process of designing hybrid video meeting systems.

In this paper, we first engage with related work from Human-Computer Interaction (HCI) on hybrid meetings and Science and Technology Studies (STS) on ways of approaching understanding shared technologies. We then turn to methods, explaining our analytic methodology of video-based analysis of conversation, the source of nature of our data, and our choice of examples. We then present micro-analyses of three episodes of common technological troubles from a single meeting that lead to practices of inclusion and exclusion. We close with design suggestions that elaborate on how ‘configuring the meeting’ may be possible for diverse social phenomena where technology is a mediator of an activity.

## Related Work

HCI and CSCW research on hybrid meetings, distributed across studies about online meetings, remote meetings, virtual meetings, distributed meetings, distributed work, virtual teams, and remote work, has focused on remote participant fidelity and equality of local and remote participation (Hollan and Stornetta 1992). The methods explored are highly varied, including gaze awareness and audio-visual support (Mukawa et al. 2005; Xu et al. 2017; Ronzhin et al. 2010; Daly-Jones et al. 1998), engagement and floor control (op den Akker et al. 2009; Dommel and Garcia-Luna-Aceves 1997; Chen 2002), social presence (Bradner and Mark 2001), and addressee prediction (op den Akker and op den Akker 2009).

Research on hybrid meetings has been conducted in contexts such as business communication (Arnfalk and Kogg 2003; Panteli and Dawson 2001), workplace studies (Townsend et al. 1998), management (Creighton and Adams 1998), education (Brooks 2010), tourism and hospitality studies (Sox et al. 2014a, 2014b) and law studies (Boros 2004). These papers address changing meeting experiences and practices with hybrid meetings as well as the structural, legal, organizational and logistical implications and requirements that come along with these types of meetings. Taken together, this research shows that no matter what technology is used, participants are motivated by their institutional goals. Meeting fidelity and equality of local and remote participation are only the means to an end.

As the technology of remote communication improves and diversifies to a considerable extent, hybrid and virtual meeting experiences vary across different working teams and workplaces. The degree of hybridity and virtuality of remote meetings raises different issues and needs to be fixed to improve meeting and team effectiveness. Compared to co-located working groups, global virtual teams, who may rarely or never meet physically, may have difficulties in building trust, common culture, and understanding. This becomes more prominent when the virtual team consists of multicultural group members, who are less interdependent in their work (Gibson and Manuel 2003, p. 64). In their case study on the usage of virtual collaboration technologies, Tan and Kondoz (2008) categorized organizational and technology barriers to conducting an efficient virtual collaboration. They found that cultural differences among virtual teams affect the equality of participation and that technical issues with video are sources of both stress and humor in different virtual meetings (Tan and Kondoz 2008).

In one of the earliest user research on distributed meetings, Yankelovich et al. (2004) list the top issues faced in remote meetings as audio, behavior and technical problems based on an internal survey in their company. While hearing issues, poor audio quality and extraneous noise are mentioned as the biggest audio problems in distributed meetings; poor meeting facilitation, insufficient preparation prior to the meeting and limitations in physical and social awareness in the sense that some participants may not see the visual materials or notice who is talking play an important role in participants’ evaluation of meeting effectiveness (Yankelovich et al. 2004, p. 421). In another multi-method user study focusing on the experiences with hybrid meetings, Yankelovich et al. (2007, p. 2790) classify further remote attendee and conference room problems in addition to audio problems and underline inequal experiences of co-located and remote participants in physical and social awareness as well as the neglect of remote participants by the in-room attendees. A more recent and extensive study on diverse remote collaboration experiences with video-conferencing and video portals at Google shows that video-based meetings even in the same company are quite diverse as the team sizes and their collaboration vary across different departments (Karis et al. 2016). However, even though improving technical features such as lighting and camera field of view (FOV) can be useful to increase awareness of remote participants regarding who is in the room, ‘primary room dominance’, which refers to the dominance of the co-located participants in the primary room, still remains as a difficult issue to be solved (Karis et al. 2016, p. 46). While remote participants can be given more prominence, enabling rapid turn-taking between co-located and remote ends to keep up with fast-moving in-room conversations is challenging (Karis et al. 2016, p. 46). Prototypes based on telepresence (Yankelovich et al. 2007) and artificial intelligence systems (Nanos and James 2013) provide a path to improving spatial and social dynamics, but such systems are many years away from ubiquitous availability.

Hybrid meetings might be considered more inclusive than co-located meetings in terms of abolishing the co-location requirement and providing flexibility in time and mobility. However, the fundamental asymmetries of video meetings (Heath and Luff 1992) broaden already given physical, social, and cultural asymmetries among different parties of the meetings (Saatçi et al. 2019). Technological breakdowns exacerbate, and are exacerbated by, such asymmetries. Prior research on technological breakdowns in meetings has come from diverse perspectives, such as activity theory (Bødker 1996) or the infrastructure lens (Star 1999). Breakdowns are vital moments in analysis of the design of the technologies because they reveal what may otherwise be invisible when technologies appear to work as intended.

Actor-Network Theory (ANT) inspired scholars in STS and HCI to re-consider the development and the use of technologies in bringing together the social and the technical and to take a symmetrical stance towards humans and artifacts. Suchman (2007) notes that the relations between humans and artifacts are not fixed. Borrowing from John Law’s (1996) concept of “labours of division”, she notes ‘artifacts are produced, reproduced, and transformed through’ their use and ‘the agencies of such artifacts do not inhere in the prescriptions themselves but rely on the skilled practices that bring them into alignment with a given case at hand’ (Suchman 2007, p. 269). Consequently, the design of a technology should not merely rely on the user or the activity, but also take into account the dynamic nature of the social phenomena and the learning experience/skilled practices that allow the stabilization of these technologies as part of broader networks.

Like every socio-technical phenomenon, hybrid meeting experiences are closely linked with the opportunities and limitations designed into meeting software and hardware. Madeleine Akrich (1992, p. 208) uses the ‘script’ metaphor to explore how designers inscribe their visions into technologies and ‘define a framework of action together with the actors and the space in which they are supposed to act’. Though used as a metaphor, such scripting is part of many design processes, where personas are created for potential users (Goh et al. 2017). However, as Bruno Latour (1992) notes, users do not necessarily act in accordance with prescribed roles. While the design process necessitates anticipation of certain users and uses, neither can be perfectly pre-determined – and, indeed, if this were so then creative uses, hacks, and workarounds would be impossible. In the same vein, Steve Woolgar (1990, p. 59) considers the design of a technology to be a process of ‘configuring’ that ‘includes defining the identity of putative users, and setting constraints upon their likely future actions’.

In this study, we aim to show how combinations of social, group, institutional, and technological dynamics throughout the meeting lead to practices of inclusion and exclusion of remote participants in order to sustain meeting continuity. These practices, we will argue, demonstrate a need to extend ‘configuration’ from users to the meeting itself.

## Methodological Background

The tools of Ethnomethodology and Conversation Analysis (EM/CA) are common analytic approaches used in HCI when there is a need to understand human action in a technological context (Suchman 2007; Heath and Luff 1992). EM/CA explores how human action at the micro-level is methodically produced (Hutchby and Wooffitt 1998; Sormani et al. 2017), rather than the narration of experience of ethnography, the interpretation of experience of surveys or interviews, or the generalization and prediction of quantitative methods. For EM/CA, the most reliable argument is one that depends on the internal warrant of understanding of turns in interaction as demonstrated sequentially by the participants in the moment, thus EM/CA argues on the basis of displayed relevance of social reasoning, rather than on the basis of distribution of cases. EM/CA allows HCI researchers to delve into how people utilize technologies as resources for interaction in ways that may be below the ‘conscious awareness’ of the people involved (Norman and Thomas 1991, p. 240), and thus improve socio-technical systems ‘around the existing features of interaction, and the pervasive abilities of users for interaction’ (Norman and Thomas 1991, p. 236).

Achieving continuity in meetings draws on aspects of conversational repair (Hutchby and Wooffitt 1998). EM/CA regards conversational turn-taking (Sacks et al. 1978), sequence organization (Schegloff and Sacks 1973), and repair (Schegloff et al. 1977) as not simply rule-following behaviors, but rather situated achievements of orderliness. CA considers repair as a fundamental part of the turn-taking system rather than a deviation or separate error-correction process (Sacks et al. 1978). As such, meeting participants’ ability to continue is predicated on the accountability of breakdown as part of the meeting talk.

## Data Collection and Analysis

**Figure 1.**
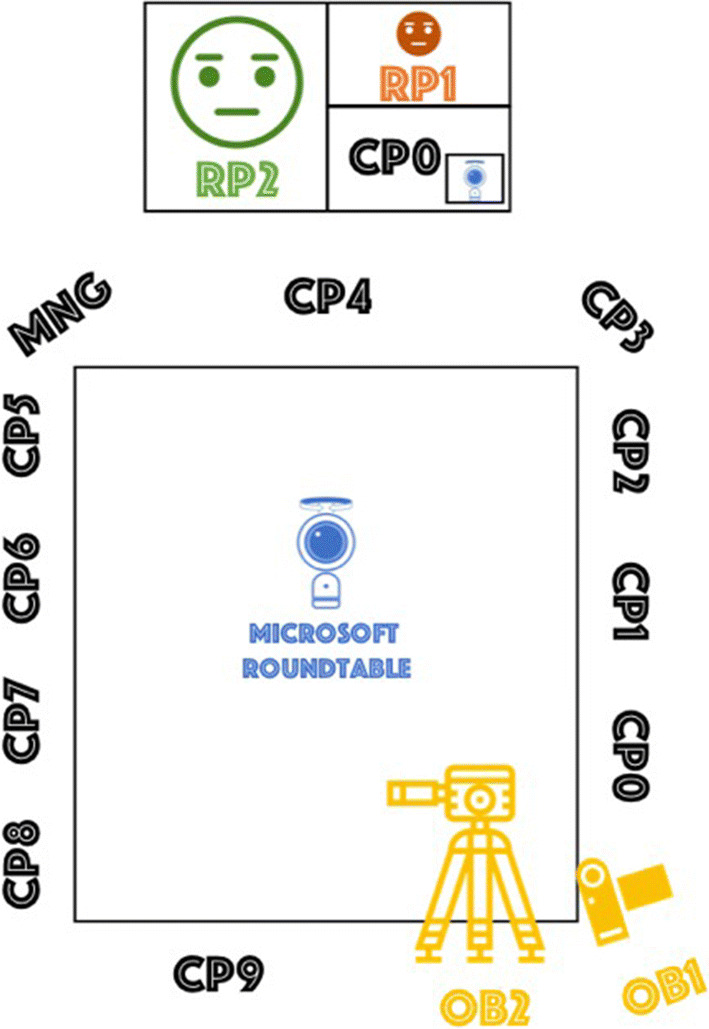
The hybrid meeting setting: Black (participants) and yellow (observers) denote individuals present in the room, green and orange figures on the screen denote remote participants. A Microsoft Roundtable 360° panoramic camera is located in the middle of the table.

The practices of inclusion and exclusion analyzed in this study were drawn from a single meeting that took place in the year of 2018 at a global software company located in the United Kingdom. We analyze three episodes in the 90-min-long video recording of this hybrid meeting. The aim of the meeting was a status update and brainstorming for representatives of the sales, marketing, and finance departments. In the meeting room there was a large screen, on which remote participants were displayed, and a table around which all the co-located participants were seated. Each participant had a personal computer, although not all were in use, and a Microsoft RoundTable 360° panoramic camera was in the middle of the table. As seen in Figure 1, a total of 11 co-located (in black) and two remote participants (in orange and green), as well as two observers (in yellow) were present.

Throughout the paper we identify the meeting participants with three characters. “CPn” refers to numbered co-located participants, with the exception of the manager, a key participant in the meeting, who is referred to as “MNG”. “RPn” refers to numbered remote participants. “OBn” refers to numbered observers (one being a co-author in this paper). On the large screen at the front of the room, the video or avatars of two remote participants (RP1 and RP2) and one co-located participant (CP0) were displayed. The reason for the appearance of CP0’s avatar on the screen in addition to remote participants is that he connected to the video call via his personal computer in order to share his screen later during the meeting. A small view of the video from the panoramic camera is shown in the lower right corner of the screen.

The meeting was recorded with a standing camera and a hand camera by the observers to capture as much interaction as possible. The positions of the cameras are shown on Figure 1. During the hybrid meeting, CP6’s computer was connected to the video call with the remote participants and Microsoft Teams was used as the video-conferencing software. CP6 moderated the meeting in the first 5 min until MNG arrived. MNG shared some details with the team and then asked for a quick status round with all participants. The status round started with reporting from CP0 and continued in the counter-clockwise direction (CP0, CP1, CP2, CP3 etc.). All three episodes are drawn from the status round.

The three episodes of inclusion and exclusion depict how meeting participants tried to repair or give up on remote participants’ inclusion and engagement, even in the same meeting, for the sake of meeting continuity. Meeting continuity, then, is the *relevancy* to which these episodes attest. For example, in the first episode we observed that the meeting was disrupted to ensure that a remote participant had video on, whereas in the third episode, the meeting was disrupted to ask a remote participant to turn off their video due to apparent network trouble. We selected these episodes from the status round section of the meeting for two reasons. First, interaction among co-located and remote participants was at its maximum in these episodes. Second, the status round itself is usefully complex because managers, moderators, and other participants are frequently attempting to refer and respond to each other. Overall, we are particularly interested in analyzing whether and how requirements and expectations can be constantly negotiated or sacrificed at both individual and collective level in order to accomplish the hybrid meeting.

The episodes were transcribed using Jefferson’s (2004) system of transcription, which aims to depict not only what is said but also how it is said sequentially by using various notation symbols^1^. Showing the details of sequential action helps untangle how each person is acting methodically in order to determine where interventions can be made to improve hybrid meeting experiences. As we were interested in analyzing both verbal and physical interactions among co-located and remote participants, we noted relevant physical details and interactions as well. After transcription, our analysis focused on sequential sociotechnical dynamics as made material through the physical and verbal actions among the participants, and how this revealed their ongoing displays of how the meeting was *supposed* to be organized and the negotiation necessary to accomplish the meeting *practically*.

## Findings

### Raising the Importance of Remote Video for Participation

**Figure 2.**
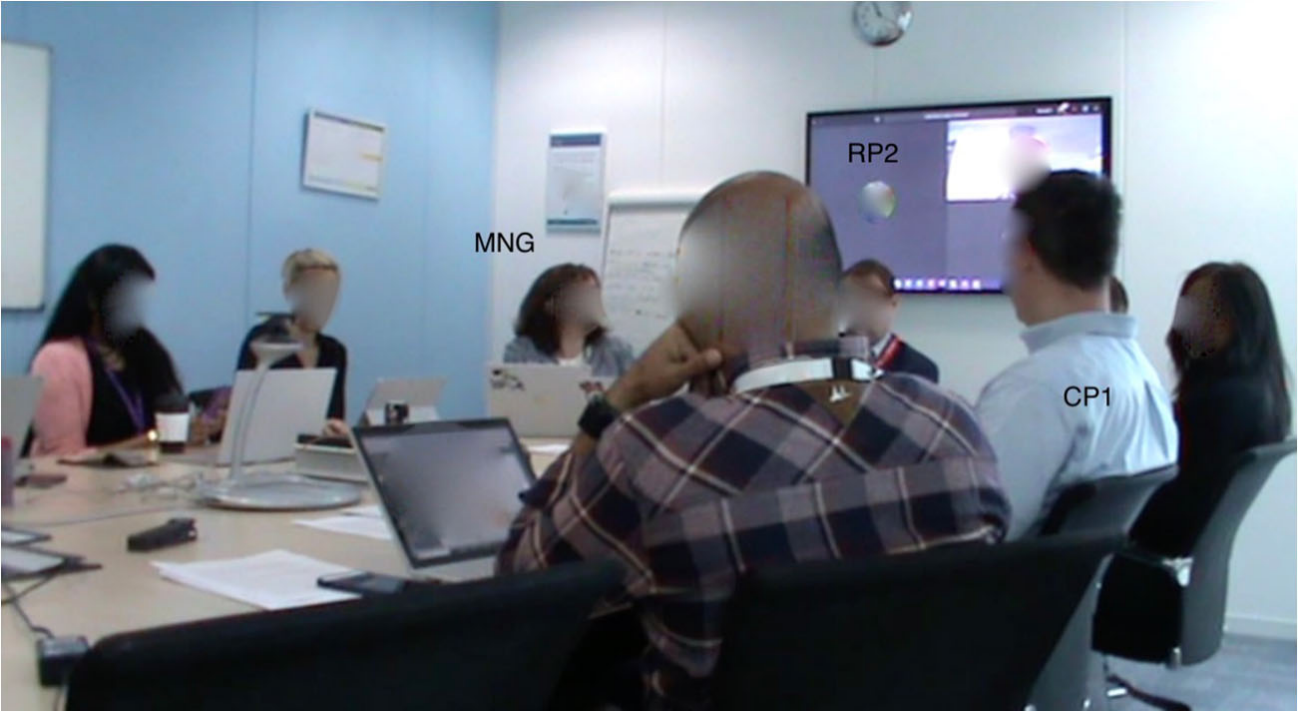
CP1 referring to RP2 by calling his name and looking at the screen expecting a reaction from him.

In the first episode, the lack of verbal reactions from a remote participant with their video off leads other meeting participants to question the participation of the remote participant and to highlight the importance of video as an indicator of presence. As CP1 begins his status update, he starts talking facing MNG but his gaze shifts to RP2’s place on the screen at the front of the room. RP2’s video is off, so his visual representation is a static image in a circle centered in his area of the grid (Microsoft Teams’ standard audio-only mode). CP1 inclines his head towards the screen and refers to RP2 claiming that he will “steal a little bit of [RP2]’s thunder” (Figure 2) (Excerpt 1: 3–6). While we might expect acknowledgement from RP2 in such a situation (verbal or non-verbal), in this case with video off, a verbal response would be possible but this is not forthcoming. CP1 continues, turning back to MNG and then looking at the screen again and raising his tone as he mentions “Friday” (the day of the deployment event he and RP2 are organizing) (Excerpt 1: 6–13). This more specific mention of a data related to their collaboration might more strongly propose that RP2 responds, but again no reaction is received. MNG and the co-located participants then shift their attention to RP2’s noticeable absence from participation. MNG turns to the screen, interrupting CP1, summoning RP2 and then teasing him: “Where’s [R]- [RP2] are you- Where is your camera on, young man?” (Excerpt 1: 18–20). This summon embeds an institutional ‘rule’ that video should be available as a means of demonstrating participation.
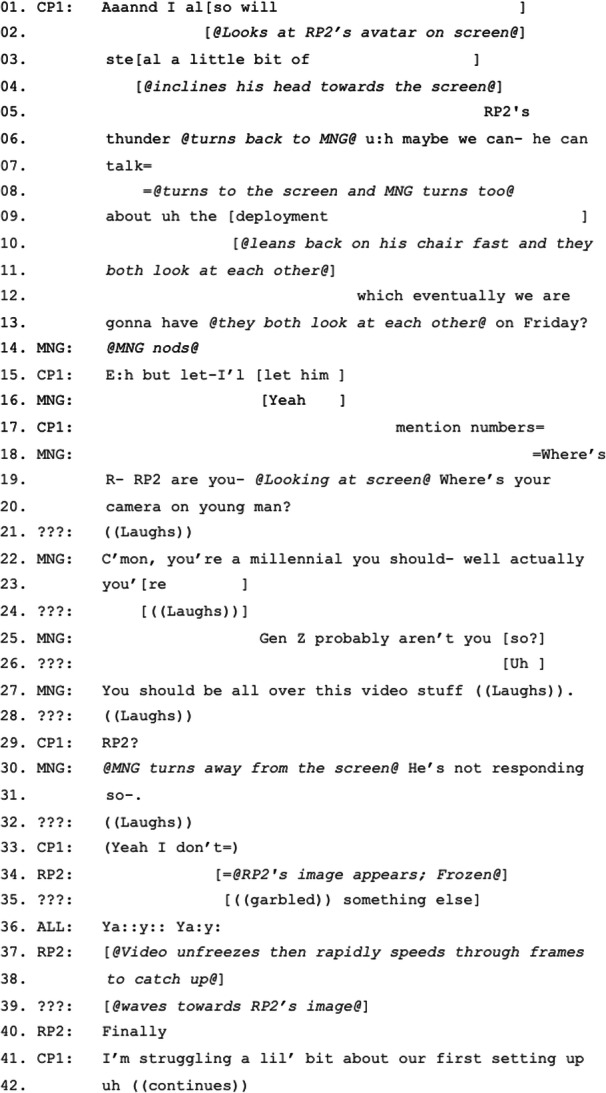


Here, MNG alludes first to RP2’s age, first calling him “young man”, then “millennial”, and finally correcting herself to say “Gen Z” stressing that he “should be all over this video stuff” (Excerpt 1: 20–27). Having received no reaction from RP2 even after all the teasing, MNG summons RP2 one last time (Excerpt 1: 29), but again receives no reaction. She then turns to the co-located participants and reports this obvious lack of response, which could function as a preliminary to proposing excluding RP2 and moving on. However, as she finishes the explanation, RP2’s video appears on the screen. The co-located participants cheer and RP2 utters his first response: “Finally”. As soon as RP2’s video is on, CP1 continues talking again, as if all is resolved.

What is particularly interesting about this episode is that a remote participant is mentioned by a co-located participant, but neither a bodily nor a verbal reaction is received. This makes MNG and, after a while, the rest of the participants question the presence or active participation of the remote participant. If his video were on, then the reason for his unresponsiveness could have been understood much easily, and the manager or the facilitator of the meeting could have focused on the connection problem directly to make sure that RP2 could hear them, saving time. However, RP2’s visible presence in audio-only mode but lack of verbal response leads MNG and the co-located participants to probe not only a potential connection problem but also the possibility that the remote participant is not paying attention. While the former is an excusable technical problem out of RP2’s hands, the latter is a more problematic moral problem for which RP2 can be held socially and institutionally accountable. This disruptive moment reveals the social dynamics that have thus far not been evident in the flow of the meeting. Rather than warning RP2 with a domineering attitude, MNG teases RP2 with the proposal that he is part of a younger generation that knows how to use technology in general and is comfortable with video in particular. By proposing that RP2 has no excuse for not turning his video on, she is also reminding him and others of the expected behavior of turning video on in hybrid meetings.

Considering this episode as a whole, there are numerous probes into whether the remote participant is still active in the meeting, beginning with CP1’s waiting and ending with MNG’s tease. Although the resolution of the breakdown is acknowledged by all participants with their cheer, it is unclear at which point the participants consider the connection and not the participant’s interest in the meeting as the cause of the problem. RP2’s succinct response “Finally” and the accompanying cheer are performative mutual agreement that there was indeed a technological problem and the elapsed time of teasing and waiting was a struggle for RP2 to find a solution, supported by the frozen video of RP2 while he is saying “Finally”. While the lack of rapport between CP1 and RP2 was the initiating element for the breakdown, when the breakdown is resolved, CP1 continues talking as soon as RP2’s video is on without involving RP2 or receiving/waiting for a response. Since RP2 has already taken enough time and attention from the meeting, neither he tries to, nor do others let him, talk more. Does the video being turned on result in a more inclusive and participatory meeting? At this point, it does not serve an inclusive function. The quest to turn on RP2’s video reveals an unspoken disciplinary requirement of the meeting that participants should be audio-visually present. However, ironically the disruption caused by the attempt to change RP2 from a noticeable absence to a visual presence is also key to the move to sideline him as an active participant, perhaps for fear of further delays. He is included only insofar as he can be accounted for as a passive participant.

### Using the Wall-Mounted Screen to Spatially Order Next Speakers

**Figure 3.**
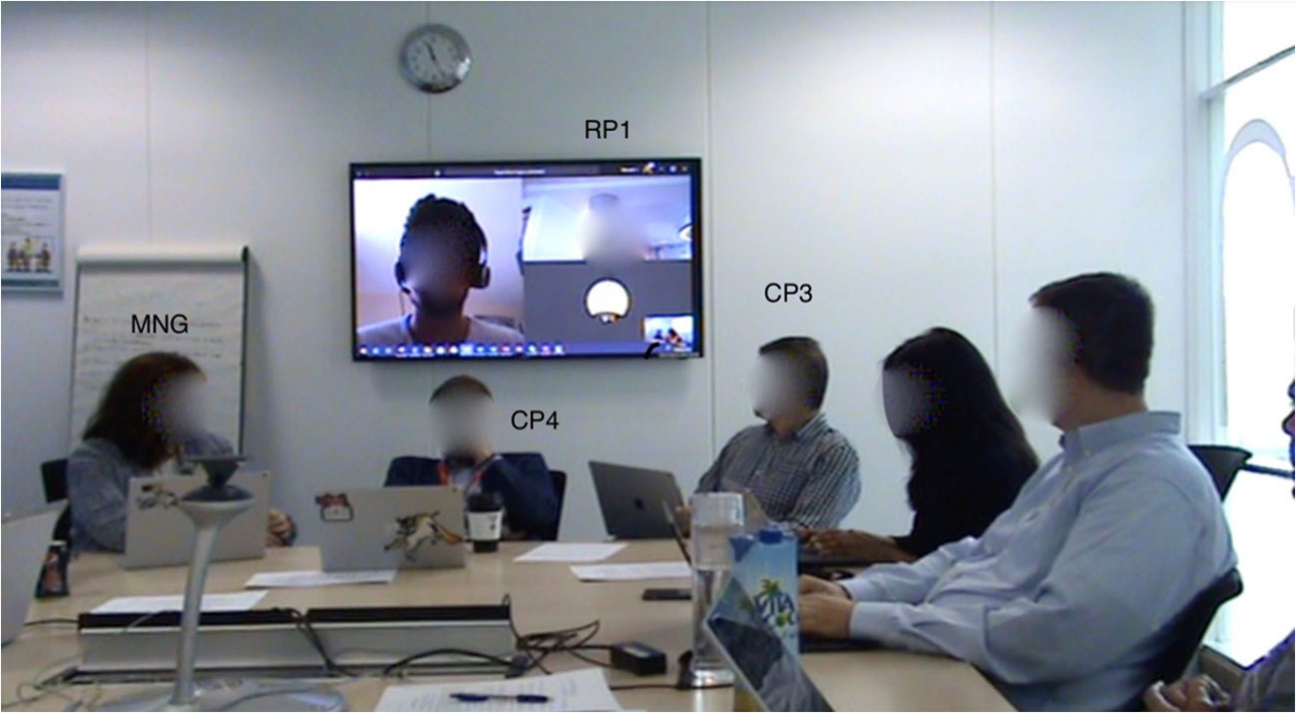
CP4 lowering himself in his chair after accepting MNG’s request to switch to the remote participants.

In the second episode, we observe an inclusive move by the manager treating the physical position of the screen and the placement of the remote participants on it as part of the spatial organization of the co-located participants at the table. When CP3 (located to the right of the screen) finishes their turn, instead of moving to CP4 (located to left of the screen), MNG suggests the continuation of spatial organization with a rhetorical question: “Should we go to the screens now?” This is followed by the verbal approval “Yeah” and nod from CP4, who would otherwise be the next speaker based on the spatial order of the table (Excerpt 2: 4–5). Further assuring the approval, the same participant lowers himself in his chair, limiting his impact as a barrier between the remote and local participants (Figure 3). Following her rhetorical question, MNG provides explanation for her reasoning by saying “Just cause it’s between you two, isn’t it?” calling for an approval also from the last speaker, pointing her fingers at both participants (Excerpt 2: 6). She then summons RP1 by calling his name, at a louder volume than she has used for participants in the room (Excerpt 2: 9). CP4 leans back and moves his chair near MNG to see the screen. To the loud summons from MNG, RP1 responds with “Here I am, hi team! Sorry I am not here today.” and continues talking (Excerpt 2: 13). After his talk ends, RP2 is summoned for his turn.
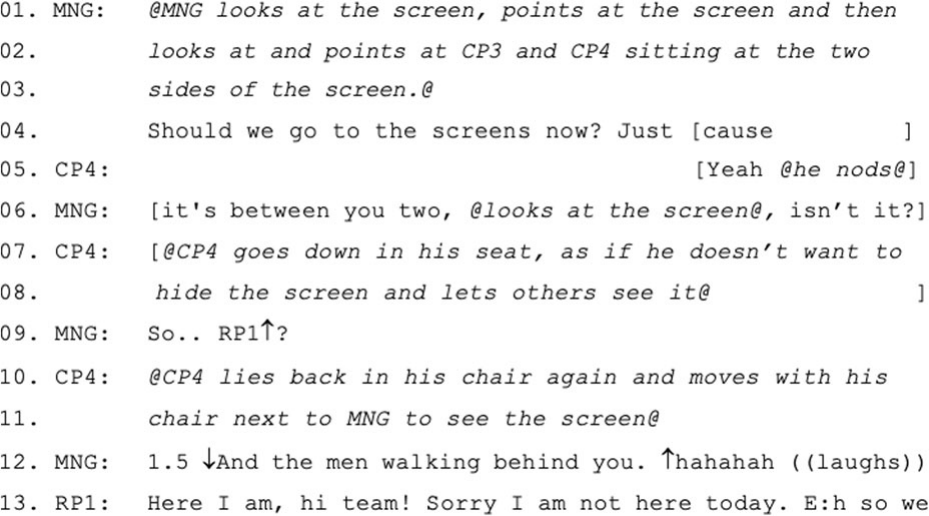

The manager makes a deliberately inclusive move to bring up the fact that the screen is located between two co-located participants and could be considered as part of the spatial organization of the local room around the table. She formulates this directive as a question, and she later justifies her reasoning, but as with the prior example of highlighting the participatory importance of video, her status as manager imbues this proposal with institutional authority and value. Had she not done so, the remote participants might have been the last ones to report. This also enhances the notion that remote participants should feel like they are part of the room with the help of technology such as the 360° camera, although it is questionable whether this is felt equally from the remote participants’ side. Furthermore, we notice that MNG uses the word “screens” referring to the two remote participants who are represented on the screen and in her following sentence, in which she justifies her reasoning, she uses the singular pronoun “it”, referring to the screen, which is physically located between CP3 and CP4. There is a possibility that she might consider moving to CP4 after RP1’s talk rather than moving to RP2 unless CP4 would change the location of his seat moving towards MNG, which would position both RP1 and RP2 in the middle of CP3 and CP4. However, it is noteworthy that remote participants are both considered as part of the spatial order based on the location of the screen compared to the co-located participants’ seats, but something about their window separation also delineating a physical separation is made relevant through the pluralization of “screens”.

Every inclusive move or technical fix which enables certain activities can still constrain others, and that is the case in this episode. While co-located participants are fully aware of the spatial order in the meeting room and can prepare themselves for their turn accordingly, remote participants do not know when they will be asked to talk. Thus, for both RP1 and RP2, it is a last-minute surprise that it will be their turn. Furthermore, unless the manager summons them, they cannot find out who should speak first as they do not know the order of their videos on the screen. Considering that participants often multitask when they have their personal computers, phones and tablets with them, distraction is even more of an issue, especially for the remote participants (Jancke et al. 2000). Lastly, it is also interesting how the manager summons RP1 to take his turn. MNG treats the remote person’s office as distant other space even while providing RP1 a reason to feel they are in the same room sharing a visible order of talk with the co-located participants. Her way of summoning also causes RP1 to respond with “Here I am”, as if she has been calling his name for a while, and finally, he shows up from another room. He then says “Sorry I am not here today”, indicating the difficulty in expressing place given the spatial-digital divide. In noting his absence “here,” he may be underlining a moral problem with remoteness. As if having remote participants is a burden to the meeting, he shows how his remote participation is not enough compared to being in the same room with the colleagues. His feeling sorry may have been exacerbated because of the delay with RP2 in the first episode.
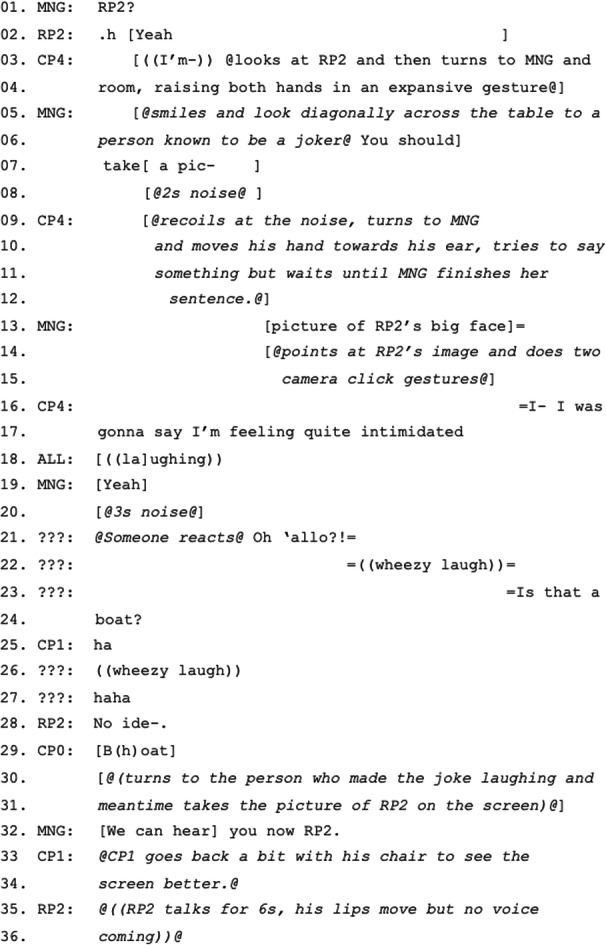


### Accounting for Letting Trouble Pass

In contrast to the first two episodes, episode 3 shows a complex set of issues leading to exclusion. MNG summons RP2 to begin his update, and while waiting after RP2’s answer, MNG looks diagonally across the table to CP0 and says “You should take a pic- picture of [RP2]’s big face”, pointing to the screen and doing ‘camera click’ gestures twice (Excerpt 3: 5–15). This treats RP2’s displayed representation on the screen as more accountable than his status as a meeting participant. CP4 begins to talk at the same time as MNG, but stops immediately waiting for her to complete her turn. MNG’s turn is interrupted by a two-second long blaring noise emanating from the room’s speaker, causing CP4 to recoil (Excerpt 3: 9). When the noise finishes MNG picks up her turn from the incomplete word (“pic-” is repeated as “picture”; Excerpt 3: 13). CP4 then reveals an exacerbated sense of technological objectification of RP2 by ‘revealing’ that he is “feeling quite intimidated” referring to RP2’s big face on the screen behind him, and all co-located participants laugh (Excerpt 3: 16–18). The blaring noise occurs again, this time for 3 s. Someone reacts with the exclamation “Oh ‘allo?!” (Excerpt 3: 21), someone else jokes “Is that a boat?” (Excerpt 3: 23–24), and everyone laughs (Excerpt 3: 25–27). RP2 attempts to start a turn responding to the joke (“No ide-”) but his sound cuts out during his second word (Excerpt 3: 28). Meanwhile, CP0 takes a photo of RP2 and laughs at the “boat” joke as well (Excerpt 3: 29–31). As the co-located participants hear RP2’s voice, MNG says to him “We can hear you” (Excerpt 3: 32), however RP2’s lips move for 6 s without sound (Excerpt 3: 35–36).
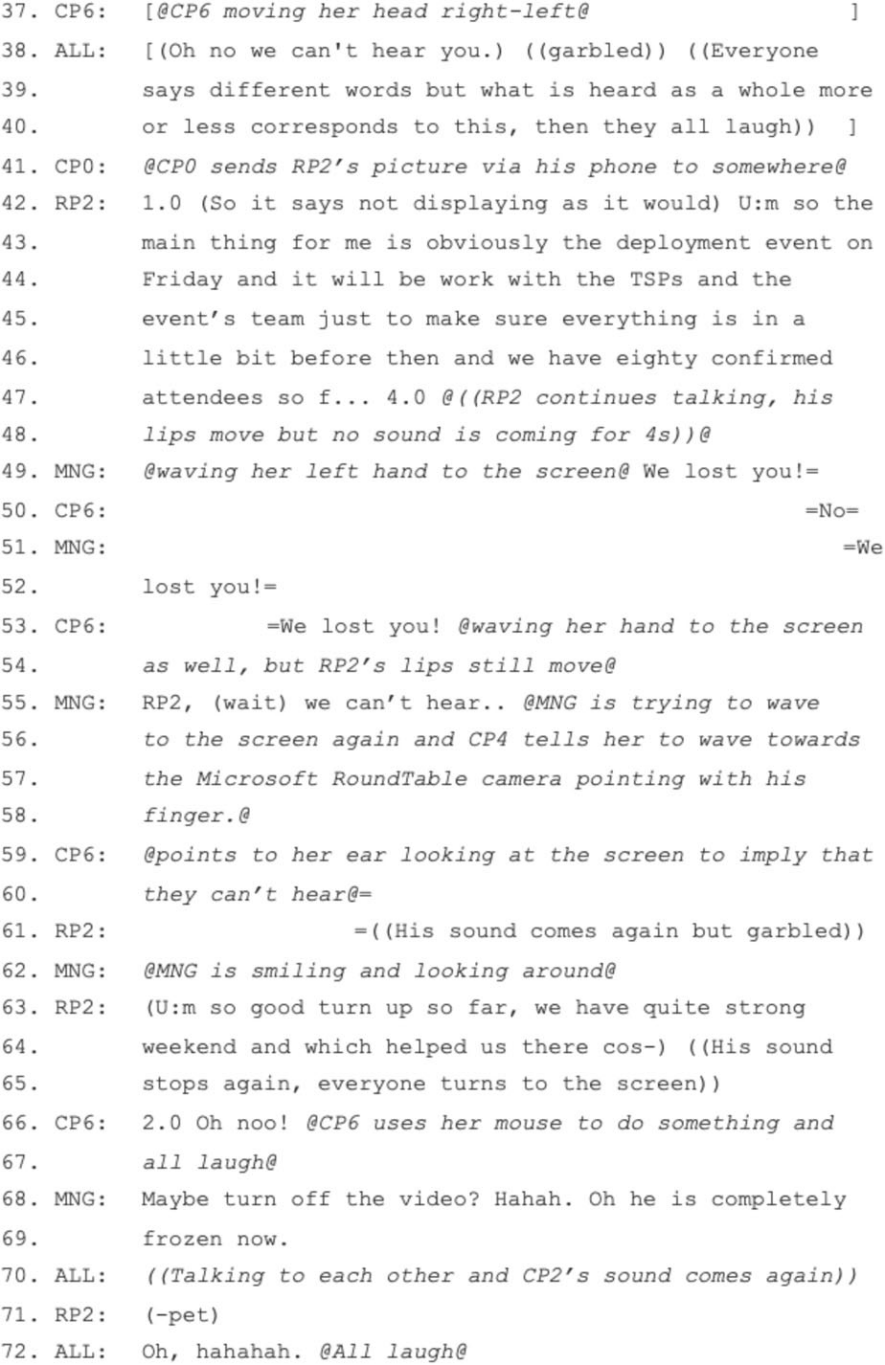


**Figure 4.**
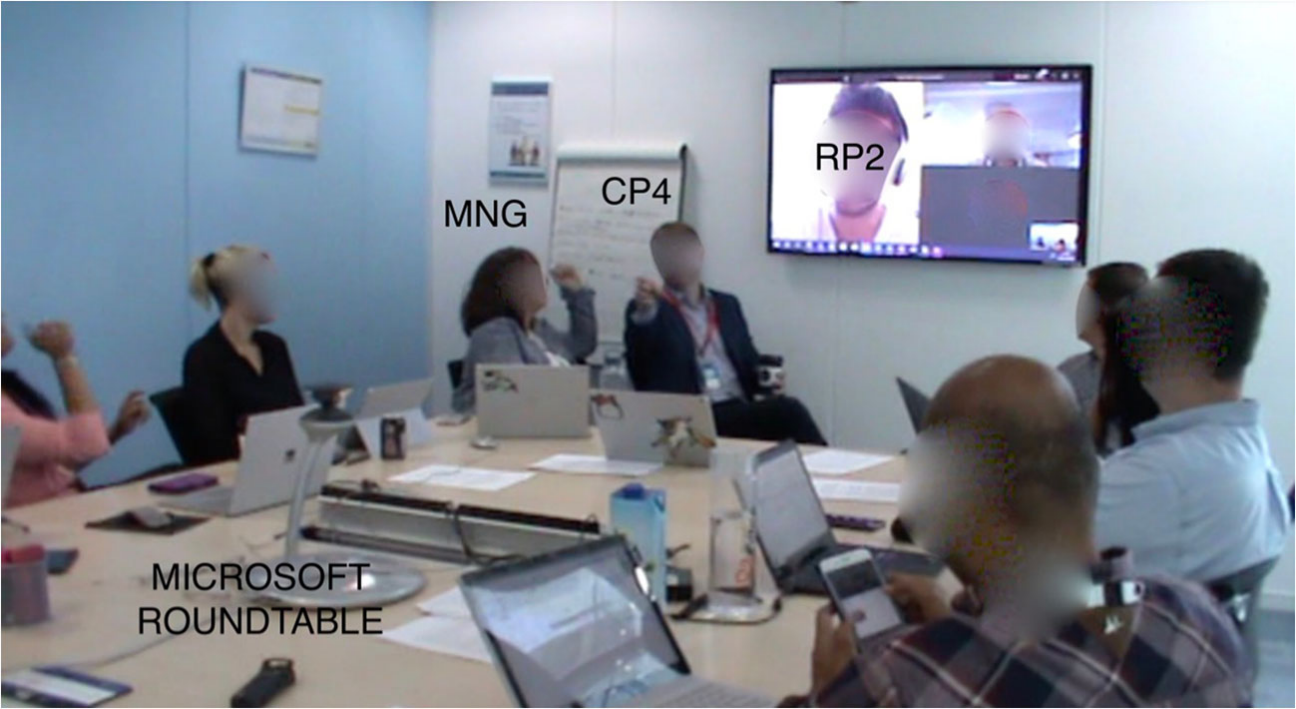
CP4 pointing to the RoundTable telling MNG to wave to it rather than to the screen.

Three seconds into RP2’s silent turn, MNG and many others call out that they cannot hear RP2 (Excerpt 4: 38–40), but his voice then returns. He talks again and can be heard for a while about a “deployment event on Friday” but after 15 s his sound stops, while his face moves (Excerpt 4: 42–48). MNG and CP6 both show their disappointment with a chorus of “We lost you” and waving to RP2 on the screen to get his attention (Excerpt 4: 50–54), but RP2 keeps talking, unaware that his voice cannot be heard. MNG is trying to wave to the screen again and CP4 tells her to wave towards the RoundTable camera, pointing with his finger (Figure 4) (Excerpt 4: 55–58). CP6 points to her ear looking at the screen to imply to RP2 that they cannot hear (Excerpt 4: 59–60). RP2’s sound comes back but then there is another 2 s of silence, which causes CP6 to react again saying “Oh noo!” and she holds her mouse to do something. In the meantime, MNG asks RP2 to turn off video, but then reports that his image is completely frozen (Excerpt 4: 68–69). RP2’s sound comes again and only the syllable “-pet” is heard. Someone says “Oh” as if he understood what he says and all laugh (Excerpt 4: 71–72). Even though it appears that RP2 regains his ability to engage (Excerpt 5: 74), MNG eventually makes a concluding move on RP2’s and the meeting’s behalf that “we got the gist” of what RP2 was attempting to report, proposing that RP2’s time on the floor has finished (Excerpt 5: 80–82). RP2 (probably) agrees (Excerpt 5: 81) and turns off his camera (Excerpt 5: 83), effectively excluding himself from continuing, and CP1 takes over immediately (Excerpt 5: 85–88). RP2 turns on his camera again, perhaps to demonstrate his continued passive presence (Excerpt 5: 89) while CP1 continues.
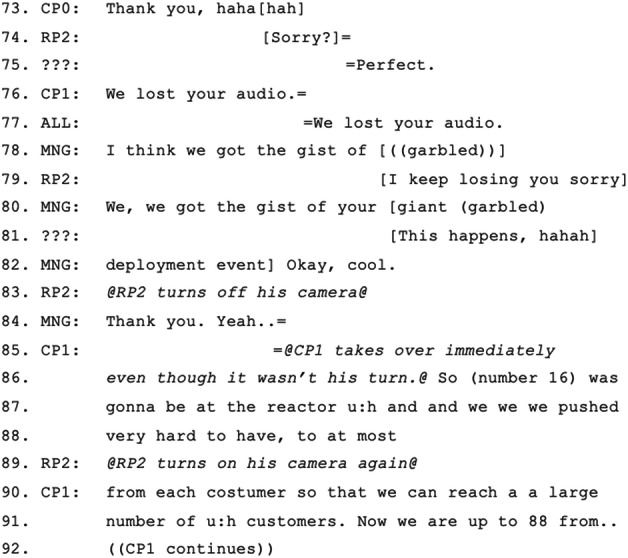

In this episode the manager enters into considerable disruption to enable RP2 to have their say, but then at some point decides that enough time has been taken up and that she needs to make an active concluding move on RP2’s and the meeting’s behalf that accounts for the conclusion as letting the trouble pass because they had “got the gist”. While having “got the gist” treats the remote participant’s report as adequate, this is a form of exclusion because it clearly marks further effort as futile. RP2 accedes to this exclusion, both by reporting technical trouble (“I keep losing you, sorry”) and by turning off his video as soon as he hears this concluding move.

Although spending one and a half minutes may not be too long for a repair depending on the importance of the status report, this is, of course, the second time that RP2 has faced technological trouble in this meeting. This may well have led MNG to believe that the same problem is recurring and cannot be solved. Even though the participants may not have fully understood or heard enough of RP2’s report, MNG does not offer or ask for alternative communication methods such as switching to a landline call or asking for a typed version through the chat. Instead, she actively shifts to the next participant. This may be a move to ensure that everyone gets equal time for their status report, but time equality is not the same as task equity. Further, although the thought of having already lost enough time due to technological breakdown might be justified, even the co-located participants do not receive equal time in the rest of the meeting. In the end, neither the equality nor equity of talk is ensured, and, as is often the case in hybrid meetings, a remote participant is partially excluded.

## Discussion

We have analyzed three episodes in which remote participants are included or excluded in a hybrid meeting in order to continue the meeting and achieve its wider goals. Similar to findings on video-calling practices of couples (Rintel 2013), we observe that participants in hybrid meetings try to overcome technical breakdowns and keep the meeting going either by fixing them, or letting them pass, or incorporating them into the interaction. While eliminating the technical pain points of remoteness at the individual level has been the main focus of video-mediated communication research for decades, we believe that a holistic approach is needed in order to better understand why many problems have still not been overcome in hybrid meetings today.

### Moving beyond Being There in Hybrid Meeting Design

Most of today’s widely-deployed video-mediated communication technologies were not designed to accommodate the needs of hybrid meetings. They draw on the legacy of videotelephony, which was designed to facilitate communication between two or more endpoints. They are not infrastructures for the ‘meeting’ of participants in the sense that they only connect the locations of participants, largely ignoring the problems of mutual engagement across different locations, especially the problem of co-located participant cliques having communicative advantages.

As Hollan and Stornetta (1992) argue, developing tools for remote communication has made the information available to all sides of meetings richer, but it may never be ‘close enough’ to replicate co-located meetings – and it is folly to try. The quality of hybrid meeting tools can always be improved, but as long as physical co-location is treated as fundamental to a meeting, remote participants will remain disadvantaged (Hollan and Stornetta 1992). Hybrid meetings often feature moments where certain actors are excluded, even when the aim is to increase inclusion, as we have shown. Thus the design question still gravitates around ‘what those tools are trying to achieve’ (Hollan and Stornetta 1992, p. 120) rather than how much their quality can be improved.

In the three moments analyzed, we draw attention to the ways in which exclusions and inclusions take place. However, our aim is to go beyond and ask how this could have been otherwise. In discussing the politics of artifacts, Akrich (1992, p. 222) suggests that ‘[t]hey may change social relations, but they also stabilize, naturalize, depoliticize, and translate these into other media’ and afterwards the processes become blackboxed in such ways that it seems ‘there was never any possibility that it could have been otherwise’. Previous research has shown that virtual meetings can function seamlessly and effectively in very large groups of over 100 remote participants, even in an audio-based setting and with participatory dynamics, because the meeting itself was configured by participants to enable broad but sparse interaction (Saatçi et al. 2019). The same study also shows that digital brainstorming platforms like Mural can lead to more inclusive and equal brainstorming sessions in hybrid meetings compared to relying on conventional brainstorming methods such as using physical whiteboards and sticky-notes, which confine idea generation only to the physical room and restrict remote participants’ engagement (Saatçi et al. 2019). That is, it may be necessary to sacrifice co-located communication and equalize the medium of meeting technology for everyone in order to achieve more inclusive hybrid meetings. We believe that after almost 30 years, there is still a need for thinking ‘beyond being there’ (Hollan and Stornetta 1992) in designing hybrid meeting tools that construct both more inclusive and more efficient meeting experiences.

### Design and Practice Suggestions for Hybrid Meetings

A rough contrast between the analyzed episodes in this paper and previously observed hybrid meetings shows that it is possible to minimize disruptions in settings where the meeting dynamics are either highly ritualized or adaptive to change. While some of this involves meeting participants learning and adopting meeting practices, some aspects of meeting dynamics can be incorporated into technological design. We will explain how this might be achieved with examples based on the episodes above.

Currently, successful hybrid meetings depend on meeting practices being adapted to/matching the configuration of the infrastructure. For example, there may be an already known order of talk or an acquired knowledge regarding what to do when certain technological breakdowns occur. In this sense, the success of the hybrid meeting often relies on the previous practice of the participants, which evolves over time to limit incongruities with what the infrastructure allows. We believe that it is possible to take an alternative route to an alignment: making the software adaptable to the meeting needs.

#### Improving Technical State Awareness

One very basic problem in hybrid meetings is that the video and audio fail to reach either or both sides. Given that the network is cause of the problem, it seems obvious that the participants could be notified about a range of network issues. In fact, early IP video-mediated communication systems often did provide this information, although often in ways most suited to experts, and as Quality of Service mechanisms improved, such notifications fell out of fashion. Network adaptation mechanisms such as scalable video codecs have also been in use for a long time (Fernández et al. 2014), but users tend to be left out of the information process. There are indications of change. WhatsApp, for example, lets both parties of a call know when either side’s battery is low. This information provides a resource for action, such as warning the other of a potential end to the conversation, or a resource for understanding that a conversation ended because one party’s battery ran out of power, even in the absence of direct communication. In hybrid meetings, technological state information could be a resource for co-located and remote participants to understand that a participant’s network may lead to the appearance of miscommunication. The issue here is to help participants head-off assumptions about presence and attention before they become problems or help resolve breakdowns as technological problems and not moral failures, because the time that it takes to resolve a technological problem, or realize it is not resolvable, may counteract potential attempts at inclusion. For example, a remote participant may not be hearing/seeing the meeting, while this does not get noticed unless a breakdown occurs or alternative communication methods are used. In the same way, it is unclear to participants how long a problem may last and whether the problem is one-sided, often due to a lack of technological information which may be readily available. In line with the design suggestions of earlier work (Yankelovich et al. 2004, p. 424), visually notifying users about audio problems, for example, could reduce problems with pursuing responses that will not be forthcoming.

#### Communicating Expected Behavior

Participants may not be aware of the expected behavior when a problem arises. In many cases, this is especially problematic from a remote participant’s perspective. For example, having video off began the disruption in Episode 1, while turning off the video for the very same participant was put forward as a solution afterwards as a way to minimize the effect of poor network connection. Whether the right behavior in this circumstance is to switch to chat or call from a landline could have been established as a choice in the planning of the meeting. In the same way, external noise from remote participants’ vicinity (such as a computer fan or nearby people talking), or even accidental noise from remote participants themselves, are both often difficult to identify in meetings with too many participants. Even with noise cancellation to reduce external noise, that the expected behavior for remote participants is to mute their audio unless speaking can be communicated before such a problem emerges, but also aided by better indications of one’s own muted status if one starts to talk or AI-based auto-unmuting using predictive pre-turn behavior tracking.

#### Communicating Meeting Formats

Meetings are more efficient when participants understand how the meeting will be structured. Components that are common in many meetings, such as status rounds, necessitate previous knowledge on how to proceed, for example, when it is the right time to speak. This is not only true for remote participants, but also for co-located ones. It is feasible to inform users of an order of talk that would be more inclusive for the remote participants, and technologically enforce this order through indicators of expected turns, while at the same time allowing flexible control over who is taking the turn when necessary.

#### Configuring the Meeting: A Hypothetical Hybrid Meeting Flow

Letting participants know what technological problems may arise, what the expected behavior is, and, how the meeting will be structured are few of many potential issues that are relevant to the hybrid meeting’s success, as we have identified in three episodes of exclusion and inclusion. While previous research has touched on these issues already, our suggested design implication is to think about these aspects and others as ‘configuring the meeting’ and not just the users. Such a configuration might be possible with questions asked to the organizer in a dialog with the service before the meeting starts or through a self-moderating system feature when there is no facilitator. For example, a hybrid meeting service could let the organizer decide and enter the details of the planned setting, format and other physical and social features of the meeting, which would allow the meeting rules, expectations, and code of conduct to be pre-set and made available to all participants equally before the meeting starts. The potential scenarios and features are endless, and of course we cannot mention all of them, so in this case we will explain what we mean by ‘configuring the meeting’ by re-imagining the status round and brainstorm meeting above as a self-moderating hybrid meeting.

In our hypothetical hybrid meeting service, the organizer configures the meeting based on the settings below before the meeting starts (Table 1). The organizer chooses the moderation setting and meeting goal(s) and format(s) for the entire meeting. The order of talk can be listed per agenda item, randomly set, or not set at all. The number of participants and their co-located and remote participation are entered. Co-located and remote participants are shown with their names as they connect to the meeting, which we show with the numbers next to the letters of C and R.

**Table 1:** . An example of configurable features set on the hybrid meeting software.

Features	Selected Configuration
Setting	*Self-moderating (No facilitator)*
Format(s)	*Status round and brainstorming*
Order of Talk	*Randomly set*
Number of Participants	*10 (6 co-located, 4 remote)*
Co-located Participants	*C1, C2, C3, C4, C5, C6*
Remote Participants	*R1, R2, R3, R4*
Allow Jump-in	*Yes*

All participants manage turn-taking through the meeting software on personal devices that they have with them. This means that while co-located participants use a shared microphone and camera to interact with the remote participants, they still connect to the meeting service on their personal devices to organize their engagement. The order of talk, which is randomly set, can be seen on personal devices with a number attached to the name of each participant. The service virtualizes ‘jump-in’ talk for both co-located and remote participants. We will explain the features we envision with a specific example.

It is C2’s turn in the status round, which is visible on everyone’s personal device. While C2 is talking, C5 wants to jump-in to ask a question. Instead of raising their physical hand, asking for permission verbally, or directly interrupting, C5 clicks the ‘jump-in’ feature on the software and then starts talking immediately. As soon as C5 is done, they ‘jump-out’, letting C2 continue talking. While C2 takes the floor again, R3 wants to jump-in directly to clarify something, which again requires R3 to click on jump-in, which directly mutes C2’s microphone (which is the shared microphone in the conference room) and unmutes R3’s microphone. When R3 is done with the fast comment, jumping-out mutes their microphone and the shared microphone is unmuted again. After C2’s report is complete, they end their section virtually by clicking ‘end turn’, which mutes their microphone and gives the floor to the next participant in the order of talk, unmuting their microphone immediately.

As we claim the need for thinking beyond being co-located and taking the diverse meeting dynamics into account, in this example, we imagine a setting where all co-located and remote participants manage turn-taking and jump-in virtually through their own devices. Turn-taking features such as raising hand, thumbs up, fast or slow talk requests as well as fast reactions for approval, already exist in distributed meeting software today, however these features are mostly used by the remote participants and mostly in virtual meetings. In line with previous research (Yankelovich et al. 2004; Yankelovich et al. 2007; Karis et al. 2016), our study demonstrates the dominance of the primary room and the inequality of co-located and remote participants. While Karis et al. (2016, p. 31) suggests forcing co-located participants to connect separately through their own devices to the meeting and in this way limiting distributed meetings to a wholly virtual setting, we believe that this is not necessary. By virtualizing some of the co-located face to face meeting features such as jumping in, training people to use these features when they are co-located, and sustaining the meeting etiquette of virtual turn-taking and jumping-in similar to self-learned manners of muting/unmuting microphones, hybrid meeting experiences might be configured to be more inclusive for all co-located and remote participants. This will, of course, require further research to be verified.

The hypothetical meeting flow we describe in this paper is just an example of configuring a hybrid meeting experience. Different distributed teams or meetings may require diverse settings and features based on their institutional needs and goals. From setting the maximum duration of talk at every jump-in to limiting the number of times a participant can jump-in, there are many physical and social features which could be set and adjusted based on every hybrid or virtual meeting’s goals and dynamics as well as the institutional rules and requirements. While such detailed configuration of hybrid meetings beforehand can also have limitations in practice, causing an increased initial overhead of planning meetings, we argue that it may empower distributed teams to discover and develop more inclusive and effective hybrid meeting set-ups, practices, and routines, enabling them to minimize the time spent on handling breakdowns in the long run.

### Moving from User-Centered Design to Meeting-Centered Design

Both commercial and research-based video-mediated communication design processes do involve versions of our suggestions for improving technological state awareness, communicating expected behavior, and communicating meeting formats. However, our aim is also to show such incorporations often forget about peoples’ need for resources to understand the meeting ecology, taking an individualized approach to potential problems. We suggest that the individual user alone should not be ‘configured’ since the meeting itself configures the participants in different ways. Thus, design of hybrid meeting technologies should consider multiplicity of the meetings and meeting needs.

Meetings are social phenomena and their dynamic nature goes beyond merely transmitting voice and video, even with sophisticated techniques to improve their apparent fidelity such as eye-tracking or rotating cameras. Being informed of technical states including potential problems, expectations, and how the meeting will be structured are configurable aspects of the meeting that not only empower the users, but are also potential factors that may increase the productivity and inclusiveness of the meeting. Designing flexible hybrid meeting services and hardware for ‘configuring the meeting’ is essential to meeting institutional goals and needs, especially in a post-COVID-19 world in which hybrid workplace attendance is likely to be the new normal.

## Conclusion

This study explores episodes of inclusion and exclusion practices relevant to common technological and turn-taking problems in hybrid meetings. Our analysis shows that hybrid meetings, like any other meeting, are shaped by the interactions and social dynamics among both participants and the technical infrastructure. Therefore, breakdowns, repairs, or passes emerging in a hybrid meeting are diverse and situational not only in every meeting, but also in every particular episode. We propose that in order to have more inclusive, democratic, and participatory hybrid meetings, there is a strong need for design solutions that are flexibly configurable according to the specific needs, goals, and dynamics of each meeting. This requires treating ‘configuring the meeting’ as the situational phenomenon at the center of the hybrid meeting design rather than an essentialist ‘scripting’ of the user.

## Footnotes

1 A list of most common symbols of Jefferson system of transcription can be found on this page: https://www2.le.ac.uk/departments/psychology/research/child-mental-health/cara-1/faqs/jefferson.
